# Clinical and Epidemiological Characteristics of Stroke in North Sumatra: A Multicenter Hospital‐Based Study

**DOI:** 10.1155/srat/7644628

**Published:** 2026-11-04

**Authors:** Fasihah Irfani Fitri, Alfansuri Kadri, Chairil Amin Batubara, Prima Heptayana Nainggolan, Oriza Novita, Etiya Ekayana, Muhammad Gandhi Sastrawan, Neni Nurchalida, Ika Mariana Gultom, Carina Shelia Puspitasari, Saulina Sembiring, Adi Putra, Petrus Nilwan Ginting, Irina Kemala Nasution, Annisa Ismira, Lisbeth Meilina Sitanggang, Meiriani Keliat, Minar Aritonang, Octaviasari Agatha Dachi, Erlina Theovani Damanik, Cut Aria Arina, Aldy Safruddin Rambe, Indonesian Neurological Association Medan Chapter, Medan, North Sumatra, Indonesia

**Affiliations:** ^1^ Department of Neurology Faculty of Medicine Universitas Sumatera Utara Medan Indonesia usu.ac.id; ^2^ Department of Neurology Adam Malik Hospital Medan Indonesia; ^3^ Department of Neurology Faculty of Medicine Universitas HKBP Nommensen Medan Indonesia; ^4^ Department of Neurology Murni Teguh Memorial Hospital Medan Indonesia; ^5^ Department of Neurology Latersia Hospital Binjai Indonesia; ^6^ Department of Neurology Inanta General Hospital Padangsidimpuan Indonesia; ^7^ Department of Neurology Dr. Tengku Mansyur Hospital Tanjungbalai Indonesia; ^8^ Department of Neurology Prof. Dr. M. Ildrem Hospital Medan Indonesia; ^9^ Department of Neurology Pirngadi General Hospital Medan Indonesia; ^10^ Department of Neurology Dolok Sanggul General Hospital Dolok Sanggul Indonesia; ^11^ Department of Neurology Panyabungan General Hospital Panyabungan Indonesia; ^12^ Department of Neurology Mitra Medika Amplas Hospital Medan Indonesia; ^13^ Department of Neurology Sultan Sulaiman General Hospital Sei Rampah Indonesia; ^14^ Department of Neurology Amri Tambunan Hospital Lubuk Pakam Indonesia; ^15^ Department of Neurology Hadrianus Sinaga General Hospital Pangururan Indonesia; ^16^ Indonesian Neurological Association Medan Chapter Medan North Sumatra Indonesia

**Keywords:** epidemiology, Indonesia, mortality, multicenter study, North Sumatra, risk factors, stroke

## Abstract

**Background:**

Stroke remains a major cause of mortality and disability worldwide, with a disproportionate burden in low‐ and middle‐income countries. In Indonesia, multicenter epidemiological data from regions outside Java remain limited. This study describes the clinical and epidemiological characteristics of stroke in North Sumatra using a multicenter hospital‐based dataset.

**Methods:**

This multicenter cross‐sectional study analyzed medical record data of patients diagnosed with stroke between January and December 2024 across 25 hospitals in North Sumatra, Indonesia. Participating hospitals included public and private facilities with head computed tomography capability. Data included demographics, vascular risk factors, stroke subtype, clinical presentation, treatment patterns, complications, and in‐hospital outcomes. Descriptive analyses were performed using frequencies, percentages, means, and medians.

**Results:**

A total of 2862 patients were included. Mean age was 61.2 ± 11.4 years, with the largest proportion aged 55–64 years (35.3%); 52.5% were male. Ischemic stroke predominated (84.6%), whereas hemorrhagic stroke accounted for 15.4%. Hypertension was the most common risk factor (80.5%), followed by dyslipidemia (32.6%) and diabetes (29.8%). Hemiparesis was the most frequent presenting symptom (63.5%). Only 11.3% of patients arrived within 4.5 h of symptom onset, whereas 45.2% arrived more than 24 h after symptom onset; arrival time was undocumented or unclassified in 20.4% of patients.

**Conclusion:**

Stroke in North Sumatra is strongly associated with modifiable vascular risk factors and delayed hospital presentation. Strengthening prevention, acute stroke care systems, and regional stroke surveillance is urgently needed.

## 1. Introduction

Stroke remains one of the most devastating neurological disorders worldwide and continues to impose substantial mortality, disability, and economic burden [1]. According to the World Stroke Organization (WSO) Global Stroke Fact Sheet 2025, stroke is currently the second leading cause of death and the third leading cause of combined death and disability‐adjusted life years (DALYs) globally [2]. The Global Burden of Disease (GBD) 2021 study estimated approximately 11.9 million new stroke cases annually, with nearly 94 million stroke survivors worldwide and more than 7 million stroke‐related deaths each year [3]. The global burden of stroke has increased substantially over the past three decades. Between 1990 and 2021, incident stroke cases increased by 70%, prevalence increased by 86%, stroke‐related deaths increased by 44%, and DALYs increased by 32% [2]. This growing burden is largely driven by population aging, population growth, and the increasing prevalence of metabolic risk factors such as hypertension, obesity, diabetes mellitus, and dyslipidemia [4, 5]. Although stroke affects all countries, its burden is disproportionately concentrated in low‐ and middle‐income countries (LMICs), which account for approximately 87.2% of stroke‐related deaths and 89.4% of stroke‐related DALYs globally [2]. Access to acute stroke interventions remains highly unequal, with fewer than 5% of eligible patients globally receiving intravenous thrombolysis and major disparities in access to neuroimaging and mechanical thrombectomy services, particularly in resource‐limited settings [6]. Indonesia faces a substantial and growing stroke burden. As the fourth most populous country in the world, Indonesia reports one of the highest stroke burdens in Southeast Asia. National data indicate that stroke remains a leading cause of mortality and long‐term disability, with an estimated incidence of 158.47 cases per 100,000 population, contributing to more than 424,000 new stroke cases recorded through the national health insurance database between 2017 and 2022 [7].

According to the Indonesian Basic Health Research Survey (*Riset Kesehatan Dasar* or *RISKESDAS*), stroke prevalence increased from 7 per 1000 population in 2013 to 10.9 per 1000 population in 2018, highlighting a worsening epidemiological trend [8]. Stroke also imposes substantial economic consequences and remains one of Indonesia′s most costly catastrophic diseases, with healthcare expenditures reaching approximately IDR 5.2 trillion in 2023. Indonesia also experiences major disparities in stroke care infrastructure. The country has limited numbers of neurologists, neurointerventionalists, stroke units, and neuroimaging facilities, most of which are concentrated in Java Island [9]. These disparities may contribute to delayed diagnosis, limited access to reperfusion therapy, and poorer outcomes in provinces outside major urban centers.

Stroke can be broadly classified into ischemic and hemorrhagic subtypes. Ischemic stroke accounts for the majority of global cases, whereas hemorrhagic stroke is generally associated with higher mortality rates [2]. In Indonesia, ischemic stroke accounted for approximately 67.1% of cases in previous multicenter studies, whereas intracerebral hemorrhage accounted for 29.6% and subarachnoid hemorrhage accounted for 3.3% [9]. Several modifiable vascular risk factors contribute significantly to stroke occurrence. Hypertension remains the most important modifiable risk factor globally and nationally. Previous Indonesian studies have reported hypertension in up to 83.4% of ischemic stroke patients, followed by dyslipidemia and diabetes mellitus [10]. Smoking also remains an important contributor, particularly among Indonesian men, where tobacco consumption remains high [11]. In addition, stroke in LMICs often occurs at younger ages compared with high‐income countries, resulting in substantial productivity loss and long‐term disability burden [12].

North Sumatra is one of Indonesia′s most populous provinces, with diverse demographic characteristics and healthcare infrastructure. Despite this, comprehensive regional stroke data remain limited. Most available studies from North Sumatra have been restricted to single‐center hospitals or relatively small sample sizes [13]. One earlier multicenter study involving hospitals in North Sumatra reported a mean patient age of approximately 59 years, a predominance of ischemic stroke, and significant limitations in access to diagnostic imaging [14]. More recent studies from individual hospitals in Medan have similarly identified hypertension as the most common risk factor and ischemic stroke as the dominant subtype, but these findings may not represent the broader provincial population.

Given the increasing burden of stroke in Indonesia, updated multicenter epidemiological data from regions outside Java remain limited. This lack of contemporary regional data hinders efforts to identify high‐risk populations, evaluate stroke care delivery, and allocate healthcare resources effectively. North Sumatra faces unique challenges, including urban–rural disparities, referral barriers, and limited access to advanced stroke services. In the absence of a national stroke surveillance system, regional datasets are essential to inform policy and strengthen stroke care systems [15]. Therefore, this study is aimed at describing the epidemiological characteristics of stroke in North Sumatra using a multicenter hospital‐based dataset, including demographic profiles, vascular risk factors, stroke subtypes, clinical presentations, treatment patterns, complications, and mortality outcomes.

## 2. Methods

### 2.1. Study Design and Setting

This study employed a multicenter hospital‐based cross‐sectional design using secondary data obtained from medical records of stroke patients. The study was conducted across multiple hospitals in North Sumatra Province, Indonesia, including both public and private healthcare facilities equipped with head computed tomography (CT) scan services to support the confirmation of stroke diagnosis. The overall study period was from May 2025 to April 2026. Data collection was performed using medical records of patients diagnosed with stroke between January 2024 and December 2024.

### 2.2. Study Population

The study population consisted of all patients diagnosed with stroke who received treatment in participating hospitals during the study period. Stroke diagnosis was defined based on clinical evaluation by physicians and confirmed by neuroimaging findings from head CT scan examination. Patients were included if they had a confirmed diagnosis of stroke and had sufficient medical record data relevant to the study variables. Patients were excluded if the diagnosis of stroke was not confirmed through neuroimaging or if key information required for the analysis was missing. As this study is aimed at capturing all eligible stroke cases recorded at the 25 participating hospitals during the study period, a total sampling (census) approach was used rather than a calculated sample size based on a priori power estimation. A total of 2,862 medical records met the inclusion criteria and were included in the final analysis.

### 2.3. Variables and Measurements

Data extracted from medical records included demographic characteristics, vascular risk factors, clinical features, and clinical outcomes. Demographic variables included age and sex. Vascular risk factors included hypertension, Type 2 diabetes mellitus, heart disease, dyslipidemia, smoking history, and alcohol consumption. These variables were recorded based on their documentation in the medical record; undocumented variables were treated as missing and were not assumed to be absent. Clinical characteristics included presenting neurological symptoms, stroke onset, hospital arrival time, length of hospital stay, and medications administered or prescribed during the index hospitalization, including antiplatelet, antihypertensive, and statin therapy. Arrival time was determined from the recorded time of symptom onset and hospital arrival, when available. Stroke severity was assessed using the National Institutes of Health Stroke Scale (NIHSS) when available in the medical records.

Treatment variables were categorized according to whether the medication was given during the index hospitalization, not based on preadmission medication history. If treatment was not given, it was recorded as “not given”; when treatment status was not available in the medical record, it was recorded as “not documented.”

Clinical outcomes assessed in this study included in‐hospital mortality, complications occurring during hospitalization, and functional outcome measures when available. Treatment variables included medications administered or prescribed during the index hospitalization, including antiplatelet therapy and antihypertensive treatment, rather than medication history before admission. Functional outcome was assessed using the modified Rankin scale (mRS) at discharge when documented. Data were extracted using a standardized extraction form, with coordination among the data‐collecting teams to harmonize data collection across participating hospitals. Because this was a medical‐record study, the availability and completeness of individual variables varied across hospitals.

Education, occupation, alcohol consumption, pre‐existing heart disease, and the timing of rehabilitation/physiotherapy were not consistently available in the source medical records across all participating hospitals and were therefore not analyzed in this study; this is noted as a limitation.

### 2.4. Data Collection Procedures

Data were collected through retrospective review of hospital medical records. After obtaining ethical approval, official permission for data access was obtained from each participating hospital. Each hospital designated a neurologist or assigned clinician as a local research coordinator responsible for data extraction and communication with the central research team. Prior to data collection, coordination meetings were conducted with participating collaborators to standardize definitions of study variables and ensure consistency in the data collection procedures across study sites. Data were recorded using a standardized data collection form developed by the research team.

### 2.5. Statistical Analysis

Statistical analyses were performed using the Statistical Package for the Social Sciences (SPSS). Descriptive statistics were used to summarize patient characteristics, including demographic variables, vascular risk factors, stroke subtypes, clinical presentations, treatment patterns, complications, and clinical outcomes. Continuous variables were presented as means with standard deviations or medians with interquartile ranges, depending on data distribution, whereas categorical variables were presented as frequencies and percentages. Comparisons between ischemic and hemorrhagic stroke subtypes were performed using independent sample *t*‐tests for normally distributed continuous variables and the Mann–Whitney *U* test for continuous variables that were not normally distributed. Chi‐square tests or Fisher′s exact tests, as appropriate, were used for categorical variables. For inferential analyses, only complete observations for the variables included in each specific analysis were used; patients with missing or undocumented values for the variables required for a particular analysis were excluded from that analysis. Statistical significance was defined as a two‐tailed *p* value of < 0.05.

### 2.6. Ethical Considerations

This study was conducted in accordance with the ethical principles outlined in the Declaration of Helsinki. Ethical approval was obtained from the Ethics Committee of the Universitas Sumatera Utara (Approval No. 389/KEPK/USU/2025) prior to data collection. Because the study used secondary data from medical records, informed consent from individual patients was waived. All collected data were anonymized and handled confidentially to ensure the protection of patient privacy.

## 3. Results

A total of 2862 patients diagnosed with stroke from 25 hospitals across multiple districts and cities in North Sumatra were included in this multicenter hospital‐based study, across different settings of healthcare facilities. Most cases were reported from urban hospitals (63.7%), with the largest proportion treated in Type C hospitals (43.5%). Stroke predominantly affected older adults, particularly those aged 55–64 years (35.3%) and 65–74 years (28.5%), and was slightly more common in men (52.5%). Ischemic stroke was the predominant subtype (84.6%), with large artery atherosclerosis (29.5% of ischemic strokes) and small vessel occlusion (27.1% of ischemic strokes) being the most common documented TOAST etiologies among ischemic stroke patients (TOAST subtypes are expressed as a percentage of the ischemic stroke, *n* = 2421, not the full cohort). Only 11.3% of patients arrived within 4.5 h of symptom onset, whereas the largest proportion presented > 24–72 h after onset (32.2%). The demographic characteristics are presented in Table 1.

**Table 1:** Demographic characteristics of patients.

Characteristic	Number (percentage)
Hospital location	
Urban area	1824 (63.7)
Rural area	1038 (36.3)
Hospital type	
Referral hospital (Type A)	684 (23.9)
District hospital (Type B)	932 (32.6)
Subdistrict level Hospital (Type C)	1246 (43.5)
Sex	
Male	1502 (52.5)
Female	1360 (47.5)
Age group (years)	
15–24	13 (0.45)
25–34	38 (1.33)
35–44	168 (5.87)
45–54	502 (17.54)
55–64	1011 (35.33)
65–74	815 (28.48)
75–84	260 (9.08)
85–94	54 (1.89)
95–100	1 (0.03)
Time from symptom onset to hospital arrival	
< 4.5 h	324 (11.3)
4.5–24 h	664 (23.2)
> 24–72 h	921 (32.2)
> 72 h–5 days	186 (6.5)
> 5 days	185 (6.5)
Not documented	566 (19.8)
Other/unclassified	16 (0.6)
Stroke subtype	
Ischemic stroke	2421 (84.6)
Hemorrhagic stroke	441 (15.4)
TOAST classification (among ischemic stroke only, *n* = 2421)	
Cardioembolism	76 (3.1)
Large artery atherosclerosis	714 (29.5)
Small vessel occlusion	657 (27.1)
Stroke of other determined	103 (4.3)
Stroke of other undetermined	173 (7.1)
Not documented	698 (28.8)

*Note:* Hospital type follows the national hospital classification of the Ministry of Health, Republic of Indonesia, which grades hospitals by service capacity, specialist and subspecialist availability, and facilities. Type A hospitals are the highest level (tertiary) referral hospitals, Type B hospitals are intermediate‐level regional/district hospitals, and Type C hospitals are basic‐level hospitals serving subdistrict and surrounding communities. Descriptive labels are used alongside the Type A/B/C designations for readers unfamiliar with the Indonesian system. TOAST stands for Trial of Org 10172 in Acute Stroke Treatment.

As shown in Figure 1, reported stroke cases were concentrated in Medan City, where several participating hospitals and major referral facilities are located. As the provincial capital, Medan has a higher density of hospitals, serves as a major referral center, and hosts several study sites, which likely contributes to the higher number of reported cases. Higher numbers of reported cases were also observed in Deli Serdang and Serdang Bedagai, with a secondary concentration in Mandailing Natal. These patterns reflect the distribution of reported cases across participating facilities and should not be interpreted as differences in population‐level stroke burden. Overall, this pattern suggests that reported stroke cases are influenced not only by disease burden but also by healthcare access and facility distribution.

**Figure 1:**
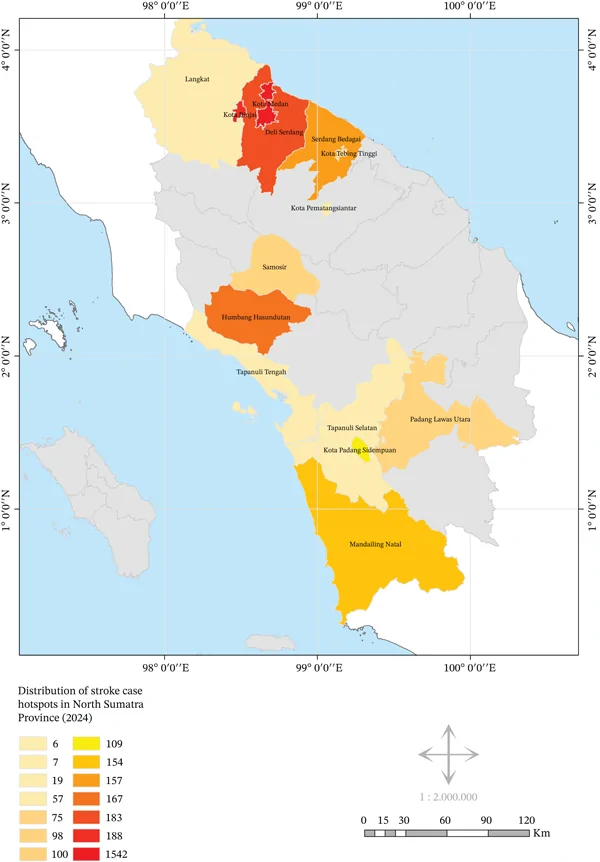
Geographic distribution of reported stroke cases across participating hospitals in North Sumatra (2024). The figure represents the distribution of cases reported by participating facilities and does not represent population‐based stroke incidence or geographic disease burden.

Baseline characteristics differed between stroke subtypes. Patients with ischemic stroke were older than those with hemorrhagic stroke (61.6 vs. 59.0 years, *p* < 0.001). Median length of hospital stay did not differ significantly between ischemic and hemorrhagic stroke patients (5 [4–6] vs. 5 [3–8] days, *p* = 0.107). Hypertension was more common in hemorrhagic stroke (87.1% vs. 79.3%), whereas diabetes mellitus (32.5% vs. 15.2%) and dyslipidemia (34.6% vs. 21.8%) were more frequent in ischemic stroke (all *p* < 0.001). No significant difference was found in sex distribution. The comparison of demographic and vascular risk factors by stroke subtype is presented in Table 2.

**Table 2:** Comparison of demographic and vascular risk factors by stroke subtype.

Characteristics	Total (*n* = 2862)	Ischemic stroke (*n* = 2421)	Hemorrhagic stroke (*n* = 441)	*p*
Age, mean ± SD (years)	61.23 ± 11.38	61.63 ± 11.06	59.04 ± 12.80	< 0.001^a^
Length of stay, median (IQR) (days)	5 (4–7)	5 (4–6)	5 (3–8)	0.107^b^
Sex				
Male, *n* (%)	1502 (52.5)	1271 (52.5)	231 (52.4)	0.964^†^
Female, *n* (%)	1360 (47.5)	1150 (47.5)	210 (47.6)	
Hypertension, *n* (%)	2305 (80.5)	1921 (79.3)	384 (87.1)	< 0.001^†^
Diabetes mellitus, *n* (%)	853 (29.8)	786 (32.5)	67 (15.2)	< 0.001^†^
Smoking history, *n* (%)	697 (24.4)	589 (24.3)	108 (24.5)	0.112^†^
Dyslipidemia, *n* (%)	934 (32.6)	838 (34.6)	96 (21.8)	< 0.001^†^
BMI category				
Normal weight	1096 (38.3)	906 (37.4)	190 (43.1)	0.334^‡^
Overweight	673 (23.5)	569 (23.5)	104 (23.6)	
Not documented	1093 (38.2)	946 (39.1)	147 (33.3)	

*Note:* Shapiro–Wilk *p* < 0.001.

^a^Independent *t*‐test.

^b^Mann–Whitney *U* test.

^†^Chi square.

^‡^BMI category was recorded in the source medical records as a binary classification (normal weight vs. overweight only, per local clinical convention) rather than the full WHO scheme, and was not documented in 38.2% of patients; the chi‐square *p* value therefore compares documented categories only (normal weight vs. overweight) between stroke subtypes and excludes undocumented cases from the test. Similarly, the chi‐square test for smoking history compares only patients with a documented smoking status (yes/no); 40.3% of patients had no documented smoking history and were excluded from this specific comparison.

Hemiparesis was the most common presenting symptom (63.5%), followed by decreased level of consciousness (20.2%), dysarthria (10.0%), and headache (6.8%). Clinical presentation differed by stroke subtype: Hemorrhagic stroke more frequently presented with decreased consciousness (51.9% vs. 14.4%), headache (15.6% vs. 5.2%), and seizures (3.9% vs. 2.2%), whereas ischemic stroke more commonly presented with focal deficits, particularly hemiparesis (68.7% vs. 35.1%) and dysarthria (11.2% vs. 3.4%) (all *p* < 0.05). Table 3 presents the clinical presentations by stroke subtype.

**Table 3:** Clinical presentations by stroke subtype.

Clinical presentation	Total (*n* = 2862) *n*(%)	Ischemic stroke (*n* = 2421) *n*(%)	Hemorrhagic stroke (*n* = 441) *n*(%)	*p*
Hemiparesis	1818 (63.5)	1663 (68.7)	155 (35.1)	< 0.001
Decreased consciousness	577 (20.2)	348 (14.4)	229 (51.9)	< 0.001
Dysarthria	285 (10.0)	270 (11.2)	15 (3.4)	< 0.001
Headache	195 (6.8)	126 (5.2)	69 (15.6)	< 0.001
Acute vestibular syndrome	131 (4.6)	128 (5.3)	3 (0.7)	< 0.001
Facial asymmetry	100 (3.5)	94 (3.9)	6 (1.4)	0.008
Hemihypesthesia	87 (3.0)	85 (3.5)	2 (0.5)	0.001
Aphasia	71 (2.5)	67 (2.8)	4 (0.9)	0.021
Seizure	70 (2.4)	53 (2.2)	17 (3.9)	0.037
Dysphagia	69 (2.4)	65 (2.7)	4 (0.9)	0.025

*Note:* One patient can present with multiple clinical presentations; chi square test.

Pneumonia was the most common in‐hospital complication (11.6%), followed by cardiac complications (5.3%) and electrolyte imbalance (3.5%). Pneumonia occurred more frequently in hemorrhagic than ischemic stroke (18.1% vs. 10.4%, *p* < 0.001). Overall, 86.3% of patients survived to discharge, whereas in‐hospital mortality was 13.7%, significantly higher in hemorrhagic stroke compared with ischemic stroke (33.7% vs. 10.1%, *p* < 0.001) (Table 4).

**Table 4:** In‐hospital complications and clinical outcomes by stroke subtype.

Variables	Total (*n* = 2862)	Ischemic stroke (*n* = 2421)	Hemorrhagic stroke (*n* = 441)	*p*
Complications				
Pneumonia	332 (11.6)	252 (10.4)	80 (18.1)	< 0.001
Urinary tract infection	21 (0.7)	18 (0.7)	3 (0.7)	0.886
Pressure ulcer	65 (2.3)	60 (2.5)	5 (1.1)	0.081
Cardiac complications	151 (5.3)	131 (5.4)	20 (4.5)	0.449
Hematologic complications	46 (1.6)	39 (1.6)	7 (1.6)	0.971
Gastrointestinal complications	28 (1.0)	22 (0.9)	6 (1.4)	0.375
Electrolyte imbalance	100 (3.5)	80 (3.3)	20 (4.5)	0.195
**Outcome**	**(n = 2747)**	**(n = 2334)**	**(n = 413)**	**< 0.001**
Survived	2372 (86.3)	2098 (89.9)	274 (66.3)	
Died	375 (13.7)	236 (10.1)	139 (33.7)	

*Note:* Mortality percentages are calculated among patients with known discharge status (*N* = 2747 overall; 2098/2334 = 89.89% ischemic; 413 hemorrhagic), excluding those with undocumented outcome (*n* = 115, 4.0%). The Outcome row (indicated in bold) shows the number of patients with known discharge status (*n* = 2747 overall; 2334 ischemic; 413 hemorrhagic), which served as the denominator for the survived and died percentages. Patients with undocumented outcome (*n* = 115, 4.0%) were excluded from this analysis. The *p* value in the Outcome row (< 0.001) indicates a statistically significant difference in outcome between ischemic and hemorrhagic stroke, with higher in‐hospital mortality in hemorrhagic stroke (33.7% vs. 10.1%). Chi‐square test.

Treatment patterns were also examined. Among all patients, aspirin monotherapy (27.7%) and aspirin plus clopidogrel (22.7%) were the most frequently used antiplatelet regimens, whereas an antiplatelet agent was explicitly withheld in 7.1% and not documented in 17.5%. Combination antihypertensive therapy was used in 35.6% of patients, followed by calcium channel blockers (24.6%). A statin was prescribed in 41.4% of patients, withheld in 42.6%, and not documented in 16.0%. These treatment patterns are summarized in Table 5.

**Table 5:** Treatment patterns during hospitalization (*N* = 2862).

Treatment pattern (*N* = 2862)	*n* (%)
Antiplatelet therapy	
Aspirin monotherapy	794 (27.7)
Aspirin + clopidogrel	650 (22.7)
Clopidogrel monotherapy	541 (18.9)
Cilostazol‐based regimen	158 (5.5)
Not given	203 (7.1)
Not documented	501 (17.5)
Other	15 (0.5)
Antihypertensive therapy	
Combination therapy	1020 (35.6)
Calcium channel blocker	705 (24.6)
Angiotensin receptor blocker	361 (12.6)
Diuretic	96 (3.4)
ACE inhibitor/beta‐blocker/other	43 (1.5)
Not given	234 (8.2)
Not documented	403 (14.1)
Statin therapy	
Given	1184 (41.4)
Not given	1220 (42.6)
Not documented	458 (16.0)

Because TOAST classification applies specifically to ischemic stroke, we further examined in‐hospital mortality across the five documented TOAST subtypes among the 2,421 ischemic stroke patients (Table 6). The “not documented” group (28.8%) was excluded from the inferential comparison because it represents missing etiological documentation rather than a biological stroke subtype. Among patients with documented TOAST classification and discharge outcomes, cardioembolic stroke carried the highest in‐hospital mortality (28.9%), followed by large artery atherosclerosis (12.6%), stroke of other undetermined etiology (8.1%), small vessel occlusion (4.9%), and stroke of other determined etiology (4.9%) (*χ* ^2^ = 61.3, df = 4, *p* < 0.001). This comparison was considered exploratory because TOAST classification was incomplete and the availability of vascular imaging, echocardiography, and prolonged cardiac rhythm monitoring varied across participating hospitals.

**Table 6:** In‐hospital mortality by TOAST subtype among ischemic stroke patients.

TOAST subtype (ischemic stroke, *n* = 2421)	*n* (% of ischemic cohort) with documented TOAST	In‐hospital deaths, *n* (% within subtype)
Cardioembolism	76 (4.4)	22 (28.9)
Large artery atherosclerosis	714 (41.4)	90 (12.6)
Small vessel occlusion	657 (38.1)	32 (4.9)
Stroke of other determined etiology	103 (6.0)	5 (4.9)
Stroke of other undetermined etiology	173 (10.0)	14 (8.1)
Total documented TOAST categories	1723 (100)	163 (9.5)

*Note:* TOAST classification was available for 1723 (71.2%) of 2421 ischemic stroke patients; 698 patients (28.8%) had no documented TOAST classification. The 698 patients without documented TOAST classification were excluded from the mortality comparison. Among the 1723 patients with documented TOAST classification, discharge outcomes were available for all five TOAST categories. Mortality percentages were calculated within each TOAST category using the corresponding category denominator (e.g., deaths among patients with cardioembolic stroke), rather than using the overall ischemic stroke population. TOAST classification was based on clinical evaluation and noncontrast CT findings; vascular imaging, echocardiography, and prolonged cardiac rhythm monitoring were not routinely available across participating hospitals. The mortality comparison was considered exploratory because incomplete etiological work‐up and variable diagnostic availability may have resulted in misclassification. Chi‐square test; *χ* ^2^ = 61.03, df = 4, *p* < 0.001.

Prehospital delay remained substantial, with only 11.3% of patients arriving within 4.5 h of symptom onset, whereas the largest proportion presented > 24–72 h after onset (32.2%). Hemorrhagic stroke patients were more likely to present within 4.5 h than ischemic stroke patients (20.9% vs. 9.6%, *p* < 0.001). Documentation of stroke severity and functional outcome was limited. Admission NIHSS was available for 75 patients (2.6%), discharge NIHSS for 54 patients, and discharge mRS for 112 patients (3.9%). Because the mRS is an ordinal scale, discharge mRS is summarized as median (IQR) rather than as a mean. Among patients with a documented discharge mRS (*n* = 112; 83 ischemic and 29 hemorrhagic stroke), the median score was 4 (IQR 3–6) overall, 4 (IQR 2–4) in ischemic stroke, and 6 (IQR 5–6) in hemorrhagic stroke. A favorable functional outcome (mRS 0–2) was observed in 25/112 patients overall (22.3%), 23/83 (27.7%) with ischemic stroke, and 2/29 (6.9%) with hemorrhagic stroke. Given the small and potentially selected subsets with available NIHSS and mRS data, these measures were presented descriptively without inferential comparison (Table 7).

**Table 7:** Hospital arrival and clinical severity by stroke subtype.

Variables	Total	Ischemic stroke	Hemorrhagic stroke	*p*
Time from symptom onset to hospital arrival				
< 4.5 h	324 (11.3)	232 (9.6)	92 (20.9)	< 0.001^†^
4.5–24 h	664 (23.2)	530 (21.9)	134 (30.4)	
> 24–72 h	921 (32.2)	818 (33.8)	103 (23.4)	
> 72 h–5 days	186 (6.5)	175 (7.2)	11 (2.5)	
> 5 days	185 (6.5)	172 (7.1)	13 (2.9)	
Not documented	566 (19.8)	479 (19.8)	87 (19.7)	
Others/unclassified	16 (0.5)	15 (0.6)	1 (0.2)	
Stroke severity				
NIHSS at admission, mean ± SD	9.71 ± 7.70 (75)	9.17 ± 7.47 (71)	19.25 ± 5.85 (4)	—
NIHSS at discharge, mean ± SD	9.48 ± 9.31 (54)	8.88 ± 9.22 (50)	17.00 ± 7.87 (4)	—
mRS at discharge, median (IQR)	4 (3–6) [112]	4 (2–4) [83]	6 (5–6) [29]	—

*Note*: Values are presented as *n* (%) for hospital arrival categories and mean ± SD for NIHSS, and median (IQR) for the modified Rankin scale (mRS). Because the mRS is an ordinal categorical scale whose grades do not represent evenly spaced clinical states, a mean score does not correspond to any defined clinical state on the 0–6 scale; median (IQR) is reported instead. Percentages for hospital arrival are calculated using the total number of patients in each stroke subtype. The “Not documented” and “Other/unclassified” categories are shown for descriptive completeness but were excluded from the chi‐square comparison of arrival‐time distribution, which was restricted to the five documented timing categories (*n* = 2280 of 2862 patients; *χ* ^2^ = 88.61, df = 4, *p* < 0.001).

^†^Chi‐square test. The number of patients with available NIHSS is shown in parentheses and in square brackets for mRS. Because NIHSS and mRS were documented in only a small and potentially selected subset of patients, no inferential statistical comparison was performed for these measures. The number of patients with available NIHSS or mRS data is shown in parentheses. Because NIHSS and mRS were documented in only a small and potentially selected subset of patients, no inferential statistical comparison was performed for these measures.

## 4. Discussion

This multicenter hospital‐based study provides the first and largest description of the clinical and epidemiological characteristics of stroke across participating hospitals in North Sumatra, involving 2862 patients. Several findings were particularly noteworthy. Ischemic stroke predominated, accounting for 84.6% of cases, whereas stroke occurred mainly among middle‐aged and older adults, with the largest proportion aged 55–64 years. Hypertension remained the leading vascular risk factor (80.5%). Substantial proportion of patients presented late, with only 11.3% arriving within 4.5 h of symptom onset and 45.2% presenting more than 24 h after symptom onset. Hemorrhagic stroke was associated with substantially higher in‐hospital mortality than ischemic stroke among patients with documented outcomes. The study also identified important gaps in clinical documentation, including incomplete etiological classification and limited documentation of stroke severity and functional outcomes. As a hospital‐based study, these findings described patients treated at participating hospitals and should not be interpreted as population‐based estimates of stroke incidence or prevalence. These findings are particularly relevant given that nearly 86% of global stroke deaths occur in LMICs, where healthcare access and preventive strategies remain uneven [2]. Within Southeast Asia, stroke contributes disproportionately to mortality and disability [16]. Indonesia reports one of the highest age‐standardized stroke mortality rates in the region at 193.3 per 100,000 person‐years, alongside one of the highest burdens of DALYs at 3382 per 100,000 people, figures surpassed only by Mongolia in the broader Asian context [17]. More recent data from the GBD 2019 study confirm that Indonesia had the highest age‐standardized stroke incidence in the WHO South‐East Asia Region (SEAR), estimated at 293.3 per 100,000, reinforcing the country′s disproportionate burden [18]. The SEAR as a whole accounts for more than 40% of global stroke mortality, and the region continues to witness an increasing stroke burden driven by aging populations and rising prevalence of vascular risk factors [18].

The mean age of patients in this study was 61.23 years, with the largest age group being 55–64 years (35.3%), followed by 65–74 years (28.5%). This pattern is consistent with Indonesian national estimates showing increasing stroke prevalence with advancing age, particularly among individuals aged 55–74 years [19]. Similar age distributions have been reported across Southeast Asia, although stroke tends to occur at younger ages in LMICs compared with high‐income countries, where incidence typically peaks after 70 years [2]. Pandian et al. (2023) reported that the mean age of stroke in South and Southeast Asia ranged between 56 and 60 years, younger than global averages, reflecting earlier exposure to vascular risk factors and suboptimal prevention strategies in the region. Notably, the INTERSTROKE study found that the proportion of young strokes (≤ 45 years) was higher in South and Southeast Asia (15.8% and 18.3%, respectively) compared with the global average of 11.8%, further underscoring the burden borne by working‐age populations in this region [18]. In our cohort, approximately 14% of patients were younger than 50 years, consistent with global estimates suggesting that 10%–15% of strokes occur in younger adults, particularly in LMIC populations [20]. This younger age distribution has important socioeconomic implications, as stroke affects individuals during their economically productive years and substantially increases long‐term caregiving burden.

Male patients slightly outnumbered female patients in our cohort, consistent with global data showing that approximately 53% of incident strokes occur in men [2]. In the context of Indonesia and the broader SEAR, this sex disparity likely reflects markedly higher smoking prevalence among men. Indonesia has one of the highest male smoking rates in Southeast Asia, reported at 76.2% among adult men, a pattern that corresponds with the high prevalence of tobacco use as a stroke risk factor documented in hospital‐based registries across the region [17, 18]. Beyond smoking, risk factor clustering tends to differ by sex in this region: Hypertension, diabetes mellitus, and tobacco use are more prevalent among men, whereas hypercholesterolemia, physical inactivity, and obesity tend to be more prevalent among women [17]. Together, these patterns likely contribute to the observed male predominance in stroke incidence and highlight the need for sex‐specific prevention strategies [21].

Ischemic stroke accounted for 84.6% of all cases, consistent with global estimates showing that approximately 80%–87% of strokes are ischemic [1, 2]. Similar findings have been reported in Indonesian hospital‐based cohorts, where ischemic stroke typically represents 67%–75% of cases [22]. Across the SEAR, GBD 2019 data confirm that ischemic stroke is the dominant subtype, with an incidence of 6.9 per 1000 compared with 5.4 per 1000 for intracerebral hemorrhage and 0.93 per 1000 for subarachnoid hemorrhage [18]. Among ischemic stroke cases in our cohort, large artery atherosclerosis and small vessel occlusion were the most common TOAST subtypes. This pattern is broadly consistent with previous reports from Asian populations, where small vessel disease and intracranial atherosclerotic disease are important mechanisms of ischemic stroke. This is consistent with findings from Harris et al. (2018), who reported that large artery atherosclerosis accounted for 59.6% of ischemic stroke cases in an Indonesian tertiary hospital cohort [10]. However, these findings should be interpreted cautiously because TOAST classification was not documented in 28.8% of ischemic stroke patients. In addition, classification was based on clinical evaluation and noncontrast CT findings, whereas vascular imaging, echocardiography, and prolonged cardiac rhythm monitoring were not routinely available across participating hospitals. Consequently, cardioembolic and large‐artery etiologies may have been under‐recognized, and misclassification cannot be excluded.

The relatively high burden of small vessel disease aligns with evidence of greater lacunar stroke prevalence among Asian populations attributable to uncontrolled hypertension [20]. Intracranial atherosclerotic disease is also a characteristic feature of ischemic stroke in the SEAR, with prevalence ranging from 20% to 53% and accounting for high rates of recurrent stroke in the region; a 10‐year registry from South India documented intracranial atherosclerosis as responsible for 78.3% of the large artery atherosclerosis subtype, contrasting sharply with Western literature, where extracranial disease dominates [18]. Additionally, cardioembolic strokes are less commonly reported in the SEAR relative to North American and European literature, a pattern potentially related to differences in the duration and extensiveness of long‐term electrocardiographic monitoring in this setting [18]. Nonetheless, the proportion of hemorrhagic strokes in our cohort and across the SEAR which was estimated at 19%–46% in various country‐level studies, remains substantially higher than the approximately 15% reported in most high‐income countries in North America and Western Europe [17, 18]. This elevated hemorrhagic stroke burden reflects the high prevalence and suboptimal control of hypertension, as well as regional dietary factors including high salt and saturated fat consumption [18]. Hemorrhagic stroke, although less frequent than ischemic stroke in our cohort, carried substantially higher in‐hospital mortality (31.5% vs. 9.7%), consistent with global evidence and underscoring its ongoing clinical importance in this setting [2]. The present study also demonstrated significant differences in in‐hospital mortality across ischemic stroke TOAST subtypes, with the highest mortality observed among patients with cardioembolic stroke. This finding is consistent with previous reports showing that cardioembolic stroke carries a substantially poorer short‐term prognosis than other ischemic stroke subtypes. The unfavorable outcome has been attributed to larger infarct size, more severe neurological impairment at presentation, and an increased likelihood of early recurrent embolism and medical complications [23]. This finding should be interpreted as exploratory rather than definitive because incomplete etiological work‐up and variable availability of vascular imaging, echocardiography, and prolonged cardiac rhythm monitoring may have affected classification. The 28.8% of patients without a documented TOAST category were excluded from the inferential comparison because “not documented” represents missing information rather than a biological stroke etiology. Therefore, the observed mortality gradient across TOAST categories should be interpreted cautiously and should not be considered definitive evidence of subtype‐specific prognosis.

Our findings are broadly consistent with the stroke epidemiology reported in Indonesia and Southeast Asia. Ischemic stroke predominated in our cohort (84.6%), whereas hypertension was the most frequently documented risk factor (80.5%), consistent with the substantial burden of vascular risk factors reported across the region [17, 18]. Stroke in South and Southeast Asia also tends to occur at younger ages than in high‐income countries, which is reflected in our cohort, where approximately 14% of patients were younger than 50 years [18, 20]. Lifestyle‐related factors, including smoking, unhealthy diet, and physical inactivity, are recognized contributors to stroke risk in Indonesia [19]. However, these factors were not sufficiently and consistently documented in our retrospective dataset and therefore cannot be identified as specific causes of the stroke burden in North Sumatra. Geographically, North Sumatra comprises densely populated urban areas as well as extensive rural and geographically dispersed regions, which may result in differences in access to healthcare and stroke services [24]. The higher number of reported cases in Medan should therefore be interpreted cautiously, as it may reflect the distribution of participating hospitals and referral patterns rather than a higher population‐level stroke burden. Population denominators and standardized catchment areas were not available in this study.

Hypertension was the most prevalent vascular risk factor (80.5%), consistent with global evidence identifying it as the leading contributor to stroke‐related DALYs worldwide [2]. This aligns with previous Indonesian and SEAR studies showing hypertension as the dominant stroke risk factor [10, 17]. In our cohort, hypertension was significantly more common among patients with hemorrhagic stroke than those with ischemic stroke (87.1% vs. 79.3%), underscoring its particularly important role in hemorrhagic stroke pathogenesis. Similarly, Arboix et al. reported that hypertension was the principal cardiovascular risk factor in spontaneous intracerebral hemorrhage and was especially associated with the atherothrombotic and lacunar subtypes of cerebral infarction [25]. Although our study did not evaluate hypertension according to individual TOAST etiological subtypes, our findings are consistent with the central role of hypertension in stroke development. The persistently high burden likely reflects inadequate detection and long‐term blood pressure control across LMICs, where hypertension remains underdiagnosed and undertreated [26]. Diabetes mellitus, dyslipidemia, and smoking were also common, highlighting the growing cardiometabolic burden and the need for stronger primary prevention strategies in Indonesia [9].

A particularly concerning finding was the substantial prehospital delay: Only 11.3% of patients arrived within 4.5 h of symptom onset, whereas most presented after 24 h. This pattern reflects multiple interacting barriers consistently documented across the SEAR. Access to timely stroke care is compromised by low public awareness of stroke symptoms, poorly organized prehospital emergency medical services (EMS), inadequate transportation infrastructure, and financial barriers [24]. In India, a SEAR country with broadly comparable healthcare challenges, only 1%–13% of stroke patients arrive by ambulance, compared with approximately 50% in high‐income countries [24]. In Thailand, where EMS systems are comparatively better structured, only 5.5%–20.5% of stroke patients utilize EMS, and only half of those transported by ambulance were appropriately prioritized to stroke‐capable centers [24]. Across the SEAR, studies have identified ignorance of warning stroke symptoms in up to 23%–48% of respondents, with more than half lacking awareness of risk factors or available treatments [24]. Sociocultural factors—including reliance on faith healers and traditional medicine—further delay presentation to formal medical care in many communities across the region [24]. In North Sumatra, where a significant proportion of the population resides in rural or periurban areas with limited access to stroke‐ready facilities, these delays are likely compounded by geographic challenges and the near‐absence of organized prehospital stroke pathways. Compared with Rambe et al. (2013), these challenges, particularly hypertension burden and delayed hospital presentation, appear to remain largely unchanged over time, reflecting the persistence of structural barriers to acute stroke care in the region [14].

Although reperfusion therapy was not directly recorded, the high proportion of late hospital arrivals suggests limited eligibility for intravenous thrombolysis. This reflects broader challenges across the SEAR, where thrombolysis use remains low due to delayed presentation, limited stroke‐ready centers, high costs, shortages of neurologists, and inadequate neuroimaging access [24]. In our cohort, the predominance of admissions to subdistrict‐level (Type C) hospitals further highlights resource limitations and the need to strengthen stroke systems through improved public awareness, emergency transport, and hub‐and‐spoke models of care [24]. The in‐hospital mortality rate of 13.1% reflects a substantial clinical burden and falls within previously reported ranges in LMIC settings [20]. This overall figure conceals a marked subtype‐specific gradient, with in‐hospital mortality of 31.5% among hemorrhagic stroke patients compared with 9.7% among ischemic stroke patients, an even wider gap than the classic 1000‐patient Barcelona cerebrovascular registry, which reported an overall in‐hospital mortality of 16.7% (28% for intracerebral hemorrhage versus 14% for ischemic stroke), and which similarly emphasized stroke subtype as a key determinant of early prognosis [27].

Pneumonia was the most common complication, particularly among hemorrhagic stroke patients, underscoring the importance of early mobilization, dysphagia screening, rehabilitation access, and organized stroke unit care [2, 28]. A major systems gap was incomplete outcome documentation, with NIHSS recorded in only 2.6% and mRS in only 3.9% of patients. This reflects broader limitations in Indonesia′s stroke surveillance infrastructure, where national registry systems remain underdeveloped [26]. These findings highlight the need for standardized data collection, routine outcome assessment, and stronger national stroke surveillance systems.

This study has several limitations. The multicenter hospital‐based design may have introduced selection bias because participating hospitals may differ in patient volume, referral patterns, diagnostic capacity, and catchment populations. The available dataset did not permit reliable reconstruction of the number of records screened or excluded at each hospital, interhospital transfers, or hospital‐specific case volumes. Therefore, duplicate counting and site heterogeneity could not be completely excluded, and the findings cannot be considered representative of the entire population of North Sumatra. As a hospital‐based registry, it may not fully capture stroke cases managed outside hospitals, particularly in rural populations with limited healthcare access. Incomplete clinical documentation may have affected stroke subtype classification, risk factor assessment, and outcome reporting, whereas prehospital factors and postdischarge outcomes were not systematically captured. TOAST subtyping relied on clinical assessment and noncontrast CT alone, without routine vascular imaging, echocardiography, or prolonged cardiac monitoring, which likely contributed to the substantial proportion of ischemic strokes with undetermined etiology and may have underestimated cardioembolic and large‐artery subtypes. The specific neurological or nonneurological cause of death was not captured in the source medical records and could therefore not be analyzed; this is an important target for future data collection. Several variables originally planned for collection, including education, occupation, alcohol consumption, pre‐existing heart disease, and the timing of rehabilitation/physiotherapy, were not consistently documented across participating hospitals and could not be analyzed. Body mass index was recorded only as a binary normal weight/overweight classification, without underweight or obese categories, and was missing in over one‐third of patients. Despite these limitations, this study provides the first large‐scale regional characterization of stroke in North Sumatra and highlights persistent gaps in prevention, acute care, and surveillance.

Future priorities include strengthening stroke awareness, improving prehospital pathways, standardizing outcome assessment (including systematic recording of cause of death and etiological work‐up for TOAST subtyping), and expanding stroke surveillance systems in Indonesia. Prospective, multicenter registries that link standardized vascular imaging and cardiac work‐up to etiology‐specific outcomes, together with evaluation of thrombolysis/thrombectomy access and organized stroke‐unit pathways, represent important priorities for future research in this region.

## 5. Conclusion

In this multicenter hospital‐based study of 2862 stroke patients in North Sumatra, ischemic stroke accounted for 84.6% of cases, and hypertension was the most frequently documented vascular risk factor (80.5%). Only 11.3% of patients arrived within 4.5 h of symptom onset, and in‐hospital mortality was substantially higher among patients with hemorrhagic stroke than ischemic stroke (31.5% vs. 9.7%).

## Funding

No funding was received for this manuscript.

## Disclosure

All scientific content, interpretation, and final editorial decisions were made solely by the authors.

## Ethics Statement

This study was conducted in accordance with the principles of the Declaration of Helsinki. Ethical approval was obtained from the Ethics Committee of Universitas Sumatera Utara Indonesia (No. 389/KEPK/USU/2025). The central ethical approval covered the multicenter study, and additional administrative approvals were obtained from participating hospitals when required.

## Consent

Patient consent was waived due to the use of secondary data obtained from existing medical records.

## Conflicts of Interest

The authors declare no conflicts of interest.

## Data Availability

The data that support the findings of this study are available from the corresponding author upon reasonable request.
